# Kinematic Analysis of Synchronized Skaters During the Off‐Ice Execution of Spiral and Spin Tasks

**DOI:** 10.1002/ejsc.12331

**Published:** 2025-07-08

**Authors:** Johanna Szenczi, Dorottya Ágoston, Rita M. Kiss, János Négyesi

**Affiliations:** ^1^ Department of Kinesiology Hungarian University of Sports Science Budapest Hungary; ^2^ Faculty of Mechanical Engineering Department of Mechatronics, Optics and Mechanical Engineering Informatics Budapest University of Technology and Economics Budapest Hungary; ^3^ Neurocognitive Research Center, Nyírő Gyula National Institute of Psychiatry, and Addictology Budapest Hungary; ^4^ CRU Hungary Ltd. Budapest Hungary

**Keywords:** biomechanics, kinesiology, motor control, team sport, youth

## Abstract

The primary objective of the present study was to examine differences and associations in joint angles and segmental swings during the off‐ice execution of one static (spiral) and one dynamic (spin) sport‐specific balance task and to determine whether motor control strategies differ when participants perform the tasks on their dominant and nondominant legs. Junior synchronized skaters (*n* = 15, age = 16.3 ± 1.5 years, years of practice: 9.8 ± 2.8 years, 10 females) performed spiral and spin tasks three times with 60 s of rest allowed between each trial. Participants' movements were captured using an optical‐based motion capture (MoCap) system that utilized 39 skin‐attached retro‐reflective markers. Our results indicate no differences in synchronized skaters' kinematic features when the spiral task is performed on their dominant versus nondominant leg (*p* > 0.05). However, the results of Spearman's correlation analyses suggest different motor control strategies between the various body segments during right‐ versus left‐leg task execution. In addition, participants produced a larger swing with their left versus right arm, regardless of whether the spiral task was performed on the dominant (39.97 ± 10.32 vs. 30.22 ± 7.80, *p* < 0.001) or the nondominant (52.88 ± 13.65 vs. 37.12 ± 9.59, *p* < 0.001) leg. Lastly, the association between the knee angle of the supporting leg and the swing of the head (*ρ* = −0.54; *p* = 0.038) suggests that the greater the knee angle of the support leg during the spins, the less compensatory head swing was needed during the task.

**Summary**

- The kinematic features of synchronized skaters did not differ between the execution of spiral tasks with the dominant and nondominant legs; however, our results suggest laterality effects on motor control strategies.
- Participants produced a larger swing with their left versus right arm, regardless of whether the spiral task was performed on their dominant or nondominant leg, likely due to sport‐specific dynamic stereotypes.
- The relationship between the angle of the supporting leg's knee and the swing of the head indicates that a larger knee angle of the support leg during the spins requires less compensatory head movement during the task.

## 1 Introduction

Synchronized skating is a growing and evolving competitive sport. The free skating program should balance all team elements with isolated movements, which should be harmoniously combined with various transitions and executed with minimal two‐footed skating. Performing not only conventional elements in synchronization with precise transitions but also executing innovative and creative elements results in special credits during competitions. Therefore, maximizing the difficulty of sport‐specific elements, including spirals, spins, circles, lines, blocks, wheels, and intersections, can be game‐changing in pursuing success at leading international championships. Previous research studies (Abbott and Hecht 2013; Dubravcic‐Simunjak et al. 2006) identified an increasing number of injuries and illnesses related to participation in synchronized skating. Singles, dance, and pair skaters are generally more agile, stronger, and more flexible than synchronized skaters; however, senior synchronized skaters tend to perform better than senior skaters in other disciplines, as indicated by higher scores, greater flexibility, larger element difficulty, and fewer segment swings (Slater et al. 2016). Therefore, skaters should likely have different strength and conditioning programs tailored to their discipline and level. Given this data and the sport's increasing popularity, establishing more sophisticated training regimen protocols and rehabilitation strategies that seek scientifically based, sport‐specific research is particularly important.

Previous neurodevelopmental studies (Pompeiano 1985; Previc 1991) suggest an enhanced role for the left leg during postural tasks, whereas the right leg concurrently generates voluntary movements due to the asymmetric prenatal development of vestibular function on the left side. Feedback from lower extremity proprioceptors shapes postural stability in standing (Allum et al. 1998). Proprioceptive function in the lower limb can be assessed through target‐matching tasks by measuring the so‐called joint position sense (JPS) (Barrett et al. 1991; Konradsen 2002). Previous research studies clarified that individuals with strong right‐side dominance consistently perceive movements more accurately in both the upper and lower extremity joints of the nondominant left versus the right‐dominant side (Goble and Brown 2007, 2008; Goble et al. 2006; Han et al. 2013; Kurian et al. 1989; Négyesi et al. 2019; Nishizawa 1991; Roy and MacKenzie 1978). Since left‐sided participants also performed a target‐matching task more accurately with their dominant left versus right knee joint (Galamb et al. 2018), it is suggested that right‐hemisphere specialization may underlie proprioceptive feedback (Goble and Brown 2007, 2008; Naito et al. 2005), irrespective of side dominance. Clinical data (Bohannon et al. 1986; Duclos et al. 2015; Perennou et al. 2008) demonstrating postural impairments, including those on the ipsilateral side to a right‐hemisphere stroke, also support this idea. Overall, the left leg is suggested to be the preferred limb for tasks requiring unipedal stability (Maki 1990). Taken together, these findings suggest that limb dominance plays a crucial role in postural control and proprioceptive feedback, highlighting the need to investigate whether synchronized skaters exhibit differential motor control strategies when performing sport‐specific balance tasks on their dominant versus nondominant leg.

Because athletes often encounter situations where the center of mass (CoM) is controlled while standing on one leg, CoM outcomes are essential in indexing standing stability (Paillard et al. 2006; Vuillerme et al. 2001). Motion capture (MoCap) is a commonly used approach for assessing the kinematic features of standing stability (Alradwan et al. 2015). Briefly, the system utilizes multiple camera recordings to calculate the trajectories of the affixed reflective markers or the position and orientation of the rigid bodies and to estimate the motions of the underlying bones, producing joint kinematics data (Cappozzo et al. 2005). Over the past few decades, lower limb joint angular kinematics via MoCap has been extensively used to analyze postural control (reviewed in (Roggio et al. 2021)). Concerning standing stability, measuring a set of variables representing change and deviation for each lower extremity joint motion time series would shed light on the kinematic features of synchronized skaters.

Overall, experimentally studying sport‐specific elements of synchronized skating is necessary to establish more sophisticated training regimen protocols and rehabilitation strategies. Although these tasks are performed with skates sliding on ice, we adapted these sport‐specific proprioceptive exercises for investigation in laboratory settings. In the present study, we aimed to determine the kinematic background mechanisms involved in the off‐ice execution of one static (spiral) and one dynamic (spin) balance task. The primary objective was to analyze the kinematic differences in synchronized skaters when performing these balance tasks on their dominant and nondominant legs. We particularly aim to examine discrepancies and associations in joint angles and segmental swings during spiral and spin tasks to determine whether motor control strategies differ based on limb dominance. Although previous studies aimed to identify the relationship between off‐ice and on‐ice performance (Bower et al. 2010), the impact of coaches' emotions on performance (Decarli et al. 2024), or to compare their agility, strength, and flexibility performance to athletes in other skating disciplines (Slater et al. 2016), to the best of our knowledge, our study is the first that aims to understand synchronized skaters' performance from a kinematic perspective. Based on the preponderance of literature suggesting right‐hemisphere specialization for standing stability in both healthy (Galamb et al. 2018; Goble and Brown 2007, 2008; Goble et al. 2006; Han et al. 2013; Kurian et al. 1989; Maki 1990; Naito et al. 2005; Négyesi et al. 2019; Nishizawa 1991; Pompeiano 1985; Previc 1991; Roy and MacKenzie 1978) and clinical (Bohannon et al. 1986; Duclos et al. 2015; Perennou et al. 2008) populations, we hypothesize that synchronized skaters will perform sport‐specific proprioceptive exercises more efficiently when standing on their left, nondominant leg.

## 2 Materials and Methods

### 2.1 Participants

Sample size calculation (G*Power 3.1.7 (Faul et al. 2007)) was performed based on a previous study (Négyesi et al. 2022) that aimed to determine the effects of side dominance on the laterality of standing stability in healthy young adults. This earlier study found significant differences between left‐ and right‐sided participants' selected kinematic variables during nondominant versus dominant unilateral stance; therefore, these effect sizes were suitable for the power analysis of the present cross‐sectional study. Considering that some effect sizes for various kinematic data of the knee joint ranged between 0.3 and 1.5, we used an effect size of 0.8, which revealed that a total sample size of 15 would be appropriate to detect significant differences between the groups, assuming a Type I error of 0.05 and a power of 0.80.

We finally recruited 15 right‐side dominant junior synchronized skaters (females: *n* = 10, age = 16.4 ± 1.65 years, height = 167.7 ± 5.7 cm, mass = 56.9 ± 3.1 kg, years of practice: 9.7 ± 3.2 years; males: *n* = 5, age = 16.2 ± 1.3 years, height = 176.2 ± 5.6 cm, mass = 64 ± 7.5 kg, years of practice: 10 ± 2 years) with no reported neurological deficits or sensorimotor impairments. Leg dominance was assessed using one‐ or two‐footed item skill tests, such as kicking a ball or stepping up onto a chair (Spry et al. 1993). After providing both verbal and written explanations of the experimental protocol, the participants and also their legal representatives signed the informed consent document in accordance with the Declaration of Helsinki. The study was approved by the University's Ethical Committee (Approval No. MTSE‐OKE‐KEB/01/2023).

### 2.2 Experimental Procedures

**FIGURE 1:**
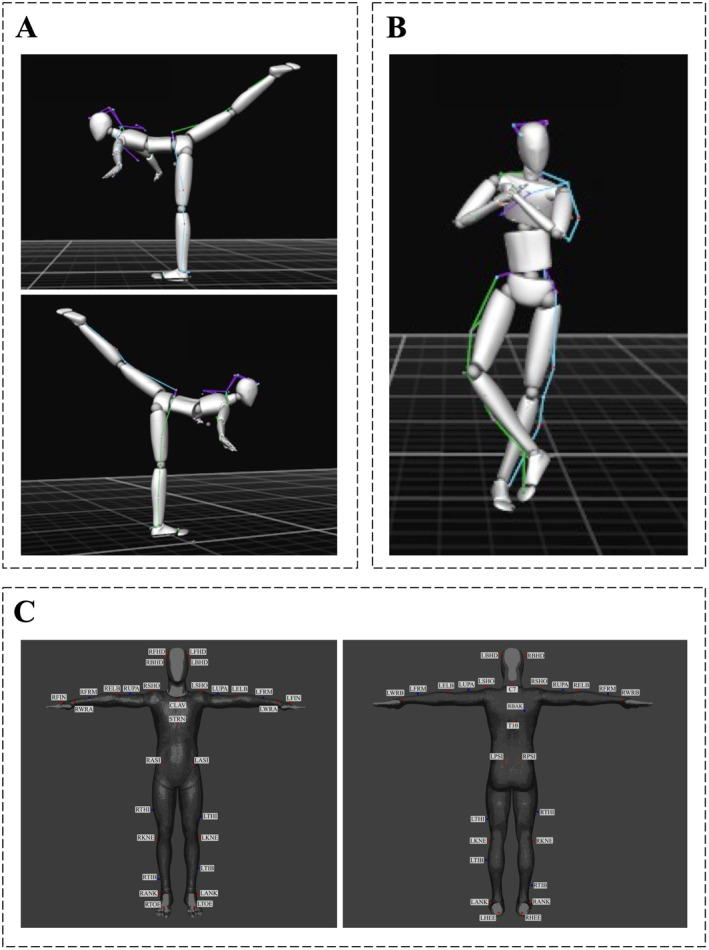
Experimental setup. (Panel A and B) 3D avatar of a representative subject during the spiral and spin tasks, respectively. (Panel C) Schematic illustration of the placement of the reflective markers for motion capture measurements. Adopted from (Pálya and Kiss 2018). *Head markers*: LHBD/RHBD, left/right posterior head; LHFD/RHFD, left/right anterior head. *Torso markers*: C7, cervical spine vertebra 7; CLAV, sternum xiphoid process; RBAK, right side of the back (not an exact location, only to aid with sides); STRN, sternum jugular notch; T10, thoracic spine vertebra 10. *Upper extremity markers*: LELB/RELB, left/right humerus lateral epicondyle; LFIN/RFIM, left/right hand second metacarpal; LFRM/RFRM left/right lower arm (not an exact location, only to aid with segments); LSHO/RSHO, left/right clavicle‐acromion joint; LUPA/RUPA, left upper arm (not an exact location, only to aid with segments); LWRA/RWRA, left/right radius styloid process; LWRB/RWRB, left/right ulna styloid process. *Lower extremity markers*: LANK/RANK, left/right lateral malleolus; LASI/RASI, left/right anterior superior iliac spine; LHEE/RHEE, left/right heel (bisection of the distal aspect of the posterior calcaneum); LKNE/RKNE, left/right knee (lateral epicondyle of the femur); LPSI/RPSI, left/right posterior superior iliac spine; LTHI/RTHI, left/right thigh (not an exact location, only to aid with sides); LTIB/RTIB, left/right shank (not an exact location, only to aid with sides); LTOE/RTOE, left/right toes (between the distal ends of the 1st and 2nd metatarsi).

After completing a sport‐specific warm‐up routine that included low‐intensity jogging and dynamic exercises for 10 min, participants performed the spiral task barefoot with each leg in a randomized order 3 times, with 60 s of rest allowed between trials. The static spiral task (Figure 1A) is a representative example of synchronized skaters' sport‐specific proprioceptive exercises, considering that this exercise is often executed alone, in pairs, or even in quadruples. Furthermore, the higher the skaters can lift their free leg, the more stable they can maintain their position, resulting in a higher score for the exercise. Participants were asked to lift their free leg as high as possible, keep their arms sideways, and maintain this position for 10 s. This hold duration was chosen because synchronized skaters typically need to maintain these positions for a similar duration during their routines. Even in off‐ice training, it is a standard practice for skaters to hold the position for 10 s, making this duration both relevant and representative of actual training and competition conditions (ISU 2024). Only successful trials were considered; therefore, if participants lost their balance, the trial was repeated after a 60‐s recovery period. Nevertheless, the frequency of failures was very low and did not differ between the dominant and nondominant legs. Specifically, in addition to the 90 successful attempts, there were eight failed attempts during the spiral task: three on the left and five on the right leg. Regarding the spin task, in addition to the 45 successful attempts, there were four failed attempts.

Given that various types of spins exist in synchronized skating, a standing spin task (Figure 1B) was also conducted with the participants' nondominant leg. The task was performed barefoot using a Tempish pirouette spinner (Tempish, Olomouc, Czech Republic), which skaters commonly use to practice movements off the ice and without skates. After three familiarization trials, participants were instructed to perform the maximum number of spins barefoot while crossing their right leg in front of their supporting left leg at the knee level with their arms positioned in front of their torso. When the skaters perform a synchronized spin in their program, a mandatory element, they all rotate in the same direction. Thus, coaches often ask skaters to practice spin tasks in only one direction. Therefore, in this study, all the athletes preferred the same direction of rotation. The task was repeated three times, with 60 s of rest allowed between each trial.

### 2.3 Motion Capture (MoCap)

Participants' movements were captured via an optical‐based MoCap system with 18 Flex13 cameras (OptiTrack, Corvallis, Oregon, USA) at a 100 fps sampling rate employing 39 skin‐attached retro‐reflective markers positioned at specific anatomical sites (Figure 1C). The best records of each participant's left and right spiral and spin were chosen for further analysis. Specifically, the best record was determined by the height of the lifted leg and the number of turns, with the highest lift and the most rotations being considered the best in the spiral and spin tasks, respectively. All records underwent post‐processing; firstly, the missing frames were interpolated using the cubic method. Thereafter, the sixth‐order Butterworth low‐pass filter was applied. All data processing was performed in MATLAB R2023b (The MathWorks Inc.; Natick, Massachusetts, USA).

### 2.4 Spiral

The spiral task resembles the arabesque movement in dance (Bronner and Shippen 2015) and ballet (C. W. Lin et al. 2024). The calculated parameters for the spiral task included the knee angle of the supporting leg, the 3D swing of the free leg, the head, torso, and both left and right arms. The spatial positions and orientations of the shank and the thigh were calculated to determine the knee angle of the supporting leg. For this purpose, the markers on the ankle (LANK or RANK) and knee (LKNE or RKNE) defined the vector of the shank. Since the marker set used did not include markers on the trochanter major, the markers on the thigh (LTHI or RTHI)—placed along the line of the femur in the sagittal plane—and the knee (LKNE or RKNE) in the sagittal plane determined the vector direction of the thigh. The vector product of these two vectors defined the angle of the knee and was calculated for every frame. The direction of the thigh is sufficient for the vectorial product to calculate the knee angle, but the actual length of the thigh is not necessary for the evaluation. The mean of the data determined the result for the knee angle of the supporting leg. As for the swings of the body segments, the positional difference between two consecutive frames was calculated. The sum of the differences during the recordings was computed and then normalized by the elapsed time. The mean of the four head markers (LFHD, RFHD, LBHD, and RBHD) defined the swing of the head, the mean of the two shoulder markers (RSHO and LSHO) defined the swing of the torso, the mean of the two wrist markers (LWRA and LWRB for the left, RWRA and RWRB for the right arm) defined the swing of the arms, and the ankle marker of the free leg (LANK—left leg, RANK—right leg) defined the swing of the leg.

### 2.5 Spin

During the spin task (Chen et al. 2019; Mapelli et al. 2013), the knee angle of the supporting leg and the angle between the vertical axis and the axis of the body were defined. The knee angle of the supporting leg was calculated in the same way as for the spiral task. The mean value of the four markers placed on the pelvis (RASI, LASI, RPSI, and LPSI) indicated one point along the body axis, whereas the mean value of the shoulders' two markers (RSHO, LSHO) defined another point of the body axis. The vector of the body's axis was calculated by subtracting the pelvis's position vector from the shoulders' mean position vector. The coordinate system is Y‐up; therefore, the unit vector v = (0, 1, 0) defines the vertical axis. The vectorial multiplication determined the angle between the two vectors and was calculated for all the frames. The mean of the data determined the result.

### 2.6 Statistical Analyses

Statistical analyses were performed using the SPSS Statistics Package (version 28.0.1, SPSS Inc., Chicago, IL, USA). All data were checked using Shapiro–Wilk's test, as well as kurtosis and skewness values and visual inspections of their histograms and QQ plots. However, since the sample size was relatively small, nonparametric Wilcoxon signed‐rank tests were employed to compare kinematic data when the spiral task was performed on the dominant versus nondominant leg. Additionally, an effect size (*r*) was calculated from the *z*‐value of the Wilcoxon signed‐rank test $\left(r=\left|z\right|/\sqrt{n}\right)$ (Fritz et al. 2012) and can be interpreted as small (*r* ≤ 0.10), medium (*r* = 0.30), or large (*r* ≥ 0.50) (Cohen 1992). In addition, Spearman's correlation was used to assess whether the height and the swing of the free leg and the knee angle of the supporting leg were associated with the swing of the head, torso, and arms during the spiral task. We also calculated the associations between the kinematic features recorded during the spin task. The correlation coefficient (*ρ*) was interpreted as follows: *ρ* < 0.20 very weak, 0.20 < *ρ* < 0.39 weak, 0.40 < *ρ* < 0.59 moderate, correlations 0.60 < *ρ* < 0.79 strong, and correlations *ρ* > 0.80 very strong (Evans 1996). Statistical significance was set at *p* < 0.05.

## 3 Results

**FIGURE 2:**
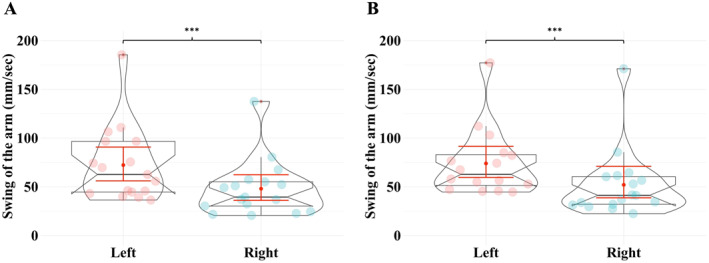
Differences between the swings of the arms during the spiral task. Swing of participants' left versus right arm, regardless if the spiral task was performed on their dominant (Panel A) or the nondominant (Panel B) leg. The violin plots (transparent color) over the boxplots represent the data distribution. The boxplots show the median, the upper, and lower quartiles and the min and max values. Red error bars within the boxplots represent the 95% confidence interval (CI) around the mean (red dot). Each data point is an individual token: the horizontal jitter is not meaningful and is only used for visualization purposes. *** indicates differences between the left and right arms in both standing conditions (*p* < 0.001). The graphs were created using RStudio software (version 2023.12.0 + 369) (Team 2020).

Raw data for each task are provided in Supporting Information S1. Statistical analyses revealed a larger swing with their left versus right arm, regardless of whether the spiral task was performed on the dominant (*Z* = −3.4; *p* < 0.001; *r* = 0.88, 39.97 ± 10.32 vs. 30.22 ± 7.80) (Figure 2A) or the nondominant (*Z* = −3.4; *p* < 0.001; *r* = 0.88, 52.88 ± 13.65 vs. 37.12 ± 9.59) (Figure 2B) leg. No other differences were found in kinematic data when participants performed the swing task with their dominant versus nondominant leg (all *p* > 0.05) (Table 1).

**TABLE 1:** Kinematic features during the spiral task with the dominant and nondominant legs.

	Height of the leg (cm)	Swing of the leg (mm/sec)	Knee angle (°)	Swing of the head (mm/sec)	Swing of the torso (mm/sec)	Swing of the Larm (mm/sec)	Swing of the Rarm (mm/sec)
D	115.3 (16.6)	59.6 (38.3)	172.8 (4.3)	29.5 (20.8)	30.0 (16.3)	74.3 (40.0)	49.1 (30.2)
ND	120.0 (16.4)	63.4 (26.4)	171.9 (5.0)	24.2 (11.4)	25.1 (11.5)	84.9 (52.9)	53.0 (37.1)
*p*‐value	0.068	0.647	0.474	0.240	0.210	0.333	0.690
Cohen's *d*	−0.511	0.121	−0.190	0.317	0.339	−0.259	−0.105

*Note:* Values are mean (SD) of each variable.

Abbreviations: D, the task was performed while standing on the dominant leg; Larm, left arm; ND, the task was performed while standing on the nondominant leg; Rarm, right arm.

Statistical analysis revealed associations between the swings of the free leg and the swing of the left (*ρ* = 0.61; *p* = 0.016) and the right (*ρ* = 0.52; *p* = 0.048) arms when the spiral exercise was performed with the participants' dominant leg. In addition to the moderate correlation between the swing of the torso and the left arm (*ρ* = 0.53; *p* = 0.041), a very strong correlation was found between the two arms when the spiral task was performed with the participants' dominant leg (*ρ* = 0.90; *p* < 0.001). On the other hand, during the execution of the nondominant leg spiral task, our results indicated a moderate correlation (*ρ* = −0.57; *p* = 0.025) between the height of the free leg and the swing of the head. In addition, the swing of the free leg was associated with the swings of the head (*ρ* = 0.58; *p* = 0.023), torso (*ρ* = 0.66; *p* = 0.008) and left arm (*ρ* = 0.59; *p* = 0.020). Furthermore, the swings of the head, torso, and arms were strongly associated with each other during the execution of the nondominant leg spiral task (each *ρ* > 0.7, each *p* < 0.005).

Regarding the spin task, Spearman's correlation analysis revealed an association between the knee angle of the supporting leg and the swing of the head (*ρ* = −0.54; *p* = 0.038), suggesting that the greater the knee angle of the support leg during the spins, the less compensatory head swing was needed during the task. In addition, the swing of the head and shoulder showed a very strong correlation (*ρ* = 0.82; *p* < 0.001) with each other.

## 4 Discussion

The primary objective of the present study was to examine the differences and associations in joint angles and segmental swings during the off‐ice execution of one static (spiral) and one dynamic (spin) sport‐specific balance task to determine whether motor control strategies differ when participants perform the tasks on their dominant and nondominant legs. Contrary to our hypothesis, participants performed the spiral task with statistically nonsignificant differences in kinematic variables between their dominant and nondominant legs. In addition, participants produced a larger swing with their left versus right arm, regardless of the performing leg. The height and swing of the free leg were associated with the swing of different body segments when the spiral task was performed with participants' dominant versus nondominant leg, suggesting that laterality affects the motor control strategies of synchronized skaters. Regarding the spin task, an inverse association was found between the knee angle of the supporting leg and the swing of the head, suggesting that a greater knee angle of the supporting leg during the spin task corresponds to a less compensatory head swing. This task was also associated with a very strong correlation between the swing of the head and the shoulder.

Due to technical limitations, we were unable to measure the kinematic features of the spiral and spin tasks in the athletes' sport‐specific environment on ice. Nevertheless, studying the aspects of sport‐related coordination, postural sway, balance, and/or postural control (Shumway‐Cook and Woollacott 2017) during the off‐ice performance of such sport‐specific balance tasks provides valuable insights for understanding the off‐ice kinematic features that may help predict skating performance. Previous studies aimed to analyze in depth the differences in balance capacity when performing various balance tasks on the dominant versus nondominant leg. According to a previous review (Paillard and Noé 2020), the influence of limb dominance on a unilateral postural balance is likely context‐dependent. In addition, muscle activation differed not only between the dominant and nondominant legs but also among left‐ and right‐sided participants during a unilateral stance (Négyesi et al. 2022) and while walking on a treadmill with patches obscuring half of the visual field (Négyesi et al. 2025). In contrast, a meta‐analysis of 46 studies (Schorderet et al. 2021) indicated that the leg's dominance does not influence unilateral balance performance. In line with this, a previous study (Promsri et al. 2020) found inconclusive results regarding a leg‐dominance effect on the coordinative structure of balancing movements. Overall, the differences between the dominant and nondominant legs during balance tasks are inconsistent and require further research to clarify whether the influence of limb dominance is context‐dependent.

Although the kinematic analysis of synchronized skaters is under‐researched, studies in other artistic sports may offer insights into between‐leg performance during balance tasks. For instance, previous findings (Jo et al. 2025) highlight differences between dancers' dominant and nondominant legs, suggesting that leg dominance should be considered in future training and performance strategies. Another study (Lin et al. 2014) indicates that both experienced and novice dancers exhibited better balance when standing on the nondominant leg during a retiré position. Moreover, the more experienced dancers had similar postural stability between their legs. Nevertheless, although both neurodevelopmental (Pompeiano 1985; Previc 1991) and behavioral (Barrett et al. 1991; Konradsen 2002) studies suggest an enhanced role for the left leg during postural tasks, our results indicated statistically nonsignificant differences in synchronized skaters’ kinematic variables when performing two sport‐specific balance tasks off‐ice with their nondominant versus dominant leg. It is suggested that right‐hemisphere specialization may underlie proprioceptive feedback (Goble and Brown 2007, 2008; Naito et al. 2005), which makes the left leg the preferred limb for tasks of unipedal stability (Maki 1990); however, the task used in the present study could be so challenging that it prevented participants from performing more accurately with their nondominant versus dominant leg. This idea is supported by the results of the correlation analyses, indicating that the swings of each body segment were strongly associated with one another during the execution of the nondominant leg spiral task. Nevertheless, only a moderate significant correlation between the swing of the torso and left arm and a very strong significant correlation between the two arms were found when the task was performed on the dominant leg. This data suggests that participants required additional compensatory movements during nondominant leg task execution. Nevertheless, from a sport‐specific perspective, it is remarkable that the height of the free leg was associated only with the swing of the head during nondominant leg task execution; however, while performing the task with the dominant leg induced such swing in the free leg that had to be compensated with the swing of each arm. This finding may highlight potential differences in the kinematic features of synchronized skaters when performing the task with their dominant versus nondominant legs.

Furthermore, the kinematic analysis of the spiral task also indicated that participants produced a larger swing of their left versus right arm, regardless of the leg used. Due to the lack of relevant information in the scientific literature on this population, this unexpected result may be explained based on our sport‐specific experiences and results from other artistic sports. In synchronized skating, maintaining proper posture is crucial for success in competitions. The optimal static position of the arms is required for a better score; thus, it is possible that participants utilized the same strategy with each leg during the spiral task of the experiment. However, considering that participants were right‐side dominant, they might have paid less attention to the left arm, which showed a more significant swing during the task, regardless of the supporting leg. Second, while executing a sport‐specific movement sequence, the left arm must perform larger movements, which may have resulted in a less stable left arm during the task. In addition, the scientific literature analyzing the role of the arms during various balance tasks suggests that the amount of arm movement activity is directly related to balance control. For example, research has shown that the dominant arm is more active in balance control, and arm movements typically occur just prior to and during the loss of balance (Shafeie et al. 2012). In line with this, results from previous studies indicate that the arms contribute to balance performance not only in the elderly (Milosevic et al. 2011) but also in the young population (Pijnappels et al. 2010), especially when the task is challenging (Muehlbauer et al. 2022).

Finally, regarding the spin task, the relationship between the angle of the supporting leg's knee and the swing of the head indicates that a larger knee angle of the support leg during the spins requires less compensatory head movement during the task. Based on our sport‐specific experience, a fully extended (180°) leg is not the optimal way to perform the spin exercise, making it challenging to correct minor deviations. Considering that the knee angle for the smallest head swing in our experiment was 172°, we hypothesize that the optimal knee angle for performing the spin task should be between 172° and 180°. The very strong correlation between the swing of the head and shoulder is expected because athletes need to keep their heads aligned with their shoulders during the spin task. To the best of our knowledge, no previous study has analyzed synchronized skaters during a spin task. However, since the pirouette task in ballet is similar to this task, we can discuss the potential underlying mechanisms of dynamic task strategies for maintaining the center of gravity over the base of support during spins based on this literature. A previous study (Tsubaki et al. 2024) showed that professional dancers produce a slightly greater inclination toward the stance leg during left rotation at the beginning of the movement (turn with double‐leg), with longer hop‐up time during single‐leg stance. In contrast, amateur dancers tended to lean forward during the left turn and single‐leg stance, resulting in a longer hop‐up time when finishing the turn on a single leg. However, another study (Lin et al. 2019) suggested that novice dancers did not adjust their posture promptly during the preparatory phase and exhibited a greater standard deviation of the COM‐COP inclination angles while making continuous postural adjustments during the ending phase. These results provide insights into the potential kinematic underlying mechanisms of spins and may explain why novice dancers perform fewer smooth movements than experienced dancers. During multiple spins, the widely used spotting technique helps reduce dizziness. However, a previous study (Schärli et al. 2024) found that spotting did not help postural stability after active rotations. Despite the results mentioned above, the underlying mechanisms during consecutive longitudinal axis rotations remain poorly understood. Therefore, a detailed analysis of whole‐body kinematics and eye‐tracking is necessary in future studies.

One limitation of our research is the relatively low sample size. Nevertheless, since the number of synchronized skaters in Hungary is limited, it would be challenging to include more subjects. Second, external factors such as differences in hamstring flexibility during the spiral task or incongruent distances between thigh markers on the left and right legs were not controlled, which could have affected, for example, the knee angle accuracy. Moreover, the limited research on the topic made it challenging to draw clear conclusions from the results. Additionally, we examined the kinematic features of synchronized skaters during the off‐ice execution of one static (spiral) and one dynamic (spin) sport‐specific balance task in a steady state, as we did not have the opportunity to use the practice space, and it was complicated to set up the MoCap. Although skaters typically practice the spin and spiral tasks in a stationary position off‐ice, the results may differ for their on‐ice performance. Additionally, future studies should clarify whether more significant inter‐limb differences would emerge if the task is performed on the ice rather than in a static position. Therefore, future studies should address this hypothesis. Nevertheless, understanding the off‐ice kinematic features could help predict skating performance by examining aspects of sport‐related coordination, postural sway, balance, and/or postural control (Shumway‐Cook and Woollacott 2017) between different body segments crucial for synchronized skating success.

## 5 Conclusion

Overall, our results indicate no differences in the kinematic features of synchronized skaters when performing the spiral task on their dominant versus nondominant leg; however, the results of the correlation analyses suggest various motor control strategies between the different body segments during right‐ versus left‐leg task execution. In addition, participants produced a larger swing with their left versus right arm, regardless of the performing leg, most likely because they also performed larger movements with their left arm during the off‐ice execution of the spiral task. Regarding the spin task, the association between the knee angle of the supporting leg and the swing of the head suggests that the greater the knee angle of the support leg during the spins, the less compensatory head swing was needed during the task. Future studies should clarify whether the kinematic features would differ from our results if the tasks were performed on ice rather than in a static position.

## Author Contributions

J.S. and J.N. designed the research. J.S. and D.A. performed the research. J.S., D.A. and J.N. analyzed the data. J.N. performed the statistical analyses. R.M.K. and J.N. directed the research. R.M.K. provided the facilities. J.S., D.A. and J.N. wrote the manuscript and all the authors have read and approved the final version.

## Ethics Statement

After giving both verbal and written explanations of the experimental protocol, participants and also their legal representative signed the informed consent document in accordance with the declaration of Helsinki. The study was approved by the University's Ethical Committee (Approval No. MTSE‐OKE‐KEB/01/2023).

## Conflicts of Interest

The authors declare no conflicts of interest.

## Supporting information

Supporting Information S1

## Data Availability

The datasets used and/or analyzed during the current study are presented within the manuscript and/or additional supporting files and are also available from the corresponding author on reasonable request.
