# Supplementary material for: Kinematic Analysis of Synchronized Skaters During the Off‐Ice Execution of Spiral and Spin Tasks
Source: Eur J Sport Sci. 2025 Jul 8;25(7):e12331. doi: 10.1002/ejsc.12331 (PMC12237585; doi:10.1002/ejsc.12331)
Supplement: Supplementary file 1 — Supporting Information S1 [file EJSC-25-e12331-s001.docx]

*Supplementary material*

**Title:** Kinematic analysis of synchronized skaters during the off-ice execution of spiral and spin tasks

**Journal name:** European Journal of Sport Science

**Authors:** Johanna Szenczi^1, a^, Dorottya Ágoston^2, a^, Rita M. Kiss^2^, János Négyesi^1, 3, 4, *^

**Affiliations:** *^1^Department of Kinesiology, Hungarian University of Sports Science, Budapest, Hungary; ^2^Faculty of Mechanical Engineering, Department of Mechatronics, Optics and Mechanical Engineering Informatics, Budapest University of Technology and Economics, Budapest, Hungary; ^3^Neurocognitive Research Center, Nyírő Gyula National Institute of Psychiatry, and Addictology, Budapest, Hungary; ^4^CRU Hungary Kft., Budapest, Hungary*

**Corresponding author:**

János Négyesi

e-mail: [negyesi.janos@tf.hu](mailto:negyesi.janos@tf.hu)

**Supplementary Data 1:** Raw data for each task
